# Cytotoxic Activity of *Piper cubeba* Extract in Breast Cancer Cell Lines

**DOI:** 10.3390/nu7042707

**Published:** 2015-04-10

**Authors:** Potchanapond Graidist, Mananya Martla, Yaowapa Sukpondma

**Affiliations:** 1 Department of Biomedical Sciences, Faculty of Medicine, Prince of Songkla University, Hat Yai, Songkhla 90110, Thailand; E-Mail: mmananya@gmail.com; 2 The Cancer Molecular Biology Excellence Research Laboratory, Prince of Songkla University, Hat Yai, Songkhla 90110, Thailand; 3 Department of Chemistry, Faculty of Science, Prince of Songkla University, Hat Yai, Songkhla 90110, Thailand; E-Mail: yaowapa.suk@psu.ac.th

**Keywords:** *Piper cubeba*, cytotoxicity, breast cancer, DNA fragmentation, apoptosis

## Abstract

This study aimed to evaluate the cytotoxicity of a crude extract of *Piper cubeba* against normal and breast cancer cell lines. To prepare the extract, *P. cubeba* seeds were ground, soaked in methanol and dichloromethane and isolated by column chromatography. Fractions were tested for cytotoxicity effects on normal fibroblast (L929), normal breast (MCF-12A) and breast cancer cell lines (MCF-7, MDA-MB-468 and MDA-MB-231). The most effective fraction was selected for DNA fragmentation assay to detect apoptotic activity. The results showed that the methanolic crude extract had a higher cytotoxic activity against MDA-MB-468 and MCF-7 than a dichloromethane crude extract. Then, the methanolic crude extract was separated into six fractions, designated A to F. Fraction C was highly active against breast cancer cell lines with an IC_50_ value less than 4 μg/mL. Therefore, Fraction C was further separated into seven fractions, CA to CG. The ^1^H-NMR profile showed that Fraction CE was long chain hydrocarbons. Moreover, Fraction CE demonstrated the highest activity against MCF-7 cells with an IC_50_ value of 2.69 ± 0.09 μg/mL and lower cytotoxicity against normal fibroblast L929 cells with an IC_50_ value of 4.17 ± 0.77 μg/mL. Finally, DNA fragmentation with a ladder pattern characteristic of apoptosis was observed in MCF-7, MDA-MB-468, MDA-MB-231 and L929 cells, but not in MCF-12A cells.

## 1. Introduction

Cancer is a major public health problem in the world. Breast cancer is the second leading cause of cancer deaths among women in the United States [1]. Chemotherapy is one of the commonly-used strategies in breast cancer treatment. This therapy is usually associated with adverse side effects, ranging from nausea to bone marrow failure [2] and development of multidrug resistance (MDR) [3]. Therefore, finding natural compounds from plants may provide an alternative cancer treatment.

In the last few decades, scientists have studied many biological properties of a number of promising plants and herbs. *Piper cubeba* L., or tailed pepper, is a plant in the family Piperaceae, genus *Piper*. This plant is a folkloric plant and has been used as a spice in many countries, including Indonesia, India, Europe (in the Middle Ages) and Morocco. The fruit has also been used for the treatment of dysentery, syphilis, gonorrhea, abdominal pain, diarrhea, enteritis and asthma [4]. *P. cubeba* has been used in Unani medicine as a protective and curative agent in various renal diseases [5]. Extracts of *P. cubeba* have various biological activities, including anti-inflammatory [6,7], anti-type IV allergic [6], anti-leishmanial [8], genotoxic [9], anti-proliferative [10] and anti-hepatitis C virus [11]. Dried *P. cubeba* contains alkaloids, lignans and terpenoids (an essential oil) [12]. Nahak and Sahu reported that *P. cubeba* ethanol crude extract has a higher antioxidant activity than the extract from another *Piper* species, *P. nigrum* extract, which could be due to a polyphenol compound. *P. cubeba* has a phenolic content which is higher than *P. nigrum* [13]. Thirteen lignans have been found in the dried fruit. Furanofuran lignans, such as cubebin, hinokinin, yatein and isoyatein are common lignans that are found in the plant genus *Piper* and also appear as a major lignans in the dried fruit of *P. cubeba* [14]. Cubebin is known to possess anti-inflammatory, analgesic [15] and anti-microbial activities [16], as well as antioxidants [17].

The aim of this present work was to determine the cytotoxic effect of *P. cubeba* crude extract on normal fibroblast (L929), normal breast (MCF-12A) and three breast cancer cell lines (MCF-7, MDA-MB-468 and MDA-MB 231). Our results showed that the Fraction CE showed a cytotoxic effect on breast cancer cell lines, and its mechanism of action seemed to include the induction of apoptosis.

## 2. Experimental Section

### 2.1. Plant Material Extraction and Isolation

*P. cubeba* seeds were acquired from Songkhla province, Thailand. Plant specimens were identified and deposited in the herbarium at the Southern Centre of Thai Traditional Medicine, Department of Pharmacognosy and Pharmaceutical Botany, Prince of Songkla University, Thailand (voucher specimen Number SKP146160302). The seeds were ground and soaked in methanol and dichloromethane for 72 h. The extract was then filtered, dried on a rotary evaporator at 45 °C, under reduced pressure. The methanolic crude extract from *P. cubeba* (33.64 grams) was separated by column chromatography on silica gel (Merck Kiesegel 60) and eluted with increasing proportions of hexane, dichloromethane and methanol. Fractions that had similar profiles on the TLC plates were pooled.

### 2.2. TLC Analysis

Thin-layer chromatography (TLC) was performed on silica gel GF_256_ (Merck). Samples of each fraction were spotted on a TLC plate. The TLC plate was developed in a beaker or closed jar. Then, a small amount of mobile phase was placed in the container. In this experiment, the solvents used as a mobile phase were hexane, dichloromethane and methanol. Fractions were separated by column chromatography and were analyzed. The same compounds were pooled and tested for any cytotoxic effects. Then, potential crude extracts were analyzed by ^1^H-NMR technique, and spectra were recorded in CDCl_3_ on a 300-MHz spectrometer (Avance 300, Bruker, Germany).

### 2.3. Cell Culture Conditions

Three breast cancer cell lines (MCF-7, MDA-MB-231, MDA-MB-468) and a normal breast cell line (MCF-12A) were obtained from ATCC (Manassas, VA, USA). Normal fibroblast cells (L929) were provided by Assoc. Prof. Dr. Jasadee Kaewsrichan (Faculty of Pharmaceutical Sciences, Prince of Songkla University, Thailand). MCF-7 cells were grown in RPMI 1640 (Invitrogen). MDA-MB-231, MDA-MB-468 and L929 cells were grown in DMEM (Invitrogen). Each medium contained 10% fetal bovine serum (Invitrogen) supplemented with 50 units/mL of penicillin (Invitrogen) and 50 μg/mL of streptomycin (Invitrogen). MCF-12A cells were grown in a 1:1 mixture of DMEM and Ham’s F12 medium (PAA Laboratories GmbH) containing 5% horse serum (Invitrogen) with 20 ng/mL human epidermal growth factor (Invitrogen), 100 ng/mL cholera toxin (Sigma), 0.01 mg/mL bovine insulin (Sigma) and 500 ng/mL hydrocortisone, 95% (Sigma). All cultures were incubated at 37 °C in an atmosphere of 5% CO_2_ and 95% air. 

### 2.4. Cytotoxic Assay

The MTT assay was used for measuring toxicity as previously described [18,19]. Briefly, cells were seeded in 96-well plates overnight. After 72 h of incubation with crude extracts, the cells were rinsed with 1X PBS and incubated with 100 μL of 0.5 mg/mL MTT at 37 °C. After 30 min of incubation, the dark blue crystals of formazan (MTT metabolites) were dissolved with 100 μL of DMSO and incubated at 37 °C for 30 min. The level of reduced MTT was determined by measuring the difference in absorbance at 570 and 650 nm using a microplate reader (Spectra Max M5, Molecular Devices). According to the U.S. NCI plant screening program, a crude extract is generally considered to have *in vitro* cytotoxic activity with an IC_50_ value ≤20 μg/mL [19].

### 2.5. DNA Fragmentation

The cells were incubated with an IC_50_ concentration of Fraction CE for 7 days. The treated cells were collected every day by trypsinization. DNA was extracted once, with an equal volume of phenol:chloroform:isoamyl alcohol (25:24:1) and once with chloroform:isoamyl alcohol (24:1). The DNA was precipitated with a two-thirds volume of cold isopropanol followed by centrifugation. The DNA pellet was washed once in 70% ethanol and resuspended in deionized water containing 0.1 mg/mL. DNA was analyzed by 1.5% agarose gel electrophoresis.

### 2.6. Statistical Analysis

Student’s *t-*test was used to analyze intergroup differences. Experiments were repeated at least three times, and data are represented as the mean ± SD. A *p*-value of less than 0.05 was considered to be statistically significant.

## 3. Results

### 3.1. The Cytotoxic Effect of Crude Extracts from P. cubeba on Breast Cancer Cell Lines

Both solvents, dichloromethane and methanol, were used to extract compounds from *P. cubeba* seeds, and MTT was used to determine their cytotoxic effects. After treatment, methanolic crude extract represented effects on MCF-7 and MDA-MB-468 with IC_50_ values of 22.31 ± 0.83 μg/mL and 21.84 ± 1.60 μg/mL, respectively, whereas the dichloromethane crude extract demonstrated a weak effect on all cell lines. Both extracts showed non-cytotoxicity to normal breast cells (Table 1). The results suggest that the optimal solvent to extract the effective compounds was methanol.

**Table 1:** Cytotoxic effects of dichloromethane and methanolic crude extracts on three breast cancer cell lines.

Compound		IC_50_ Value ± SD (μg/mL)		
	MCF-7	MDA-MB-468	MDA-MB-231	MCF-12A
Methanolic crude extract	22.31 ± 0.83	21.84 ± 1.60	65.12 ± 5.98	>80
Dichloromethane crude extract	62.20 ± 0.55	54.81 ± 0.13	35.71 ± 5.73	>80

### 3.2. The Separation of Crude Extract by Column Chromatography

**Figure 1:**
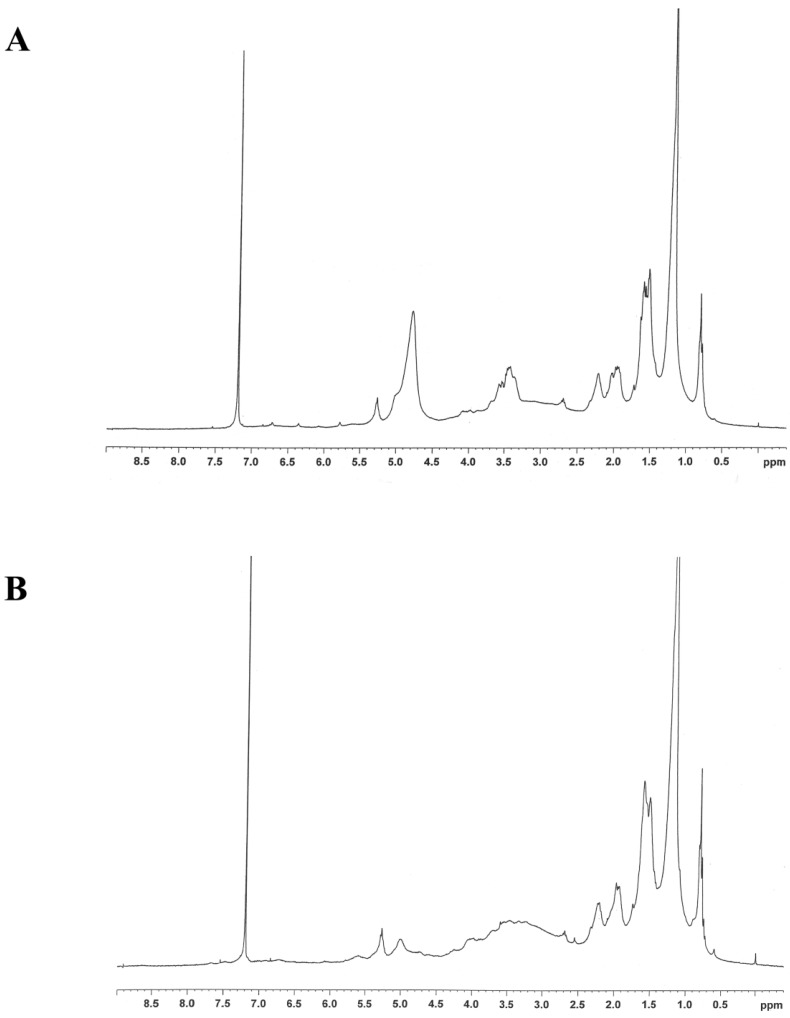
The ^1^H-NMR spectra of (**A**) Fraction C and (**B**) Fraction CE.

The structure of methanolic crude extract was analyzed by ^1^H-NMR. The ^1^H-NMR profile represented long chain hydrocarbons (data not shown). Then, the methanolic crude extract from *P. cubeba* was separated by column chromatography using silica gel and eluted with increasing proportions of hexane, dichloromethane and methanol. The fractions that had a similar profile on the TLC plate were pooled together. The separation showed six different fractions. These six fractions, labeled A to F for identification, were evaporated and tested for cytotoxic activity on breast cell lines. Fraction C proved to be the most effective fraction and was then selected to analyze the structure by ^1^H-NMR and further purified by column chromatography. The ^1^H-NMR profile showed that Fraction C was long chain hydrocarbons (Figure 1A). After separation, Fraction C showed seven different sub-fractions, labeled CA to CG. The most effective fraction was CE, and its ^1^H-NMR profile indicated the presence of long chain hydrocarbons (Figure 1B).

### 3.3. Cytotoxicity Effect of the Fractions Separated from P. cubeba

**Figure 2:**
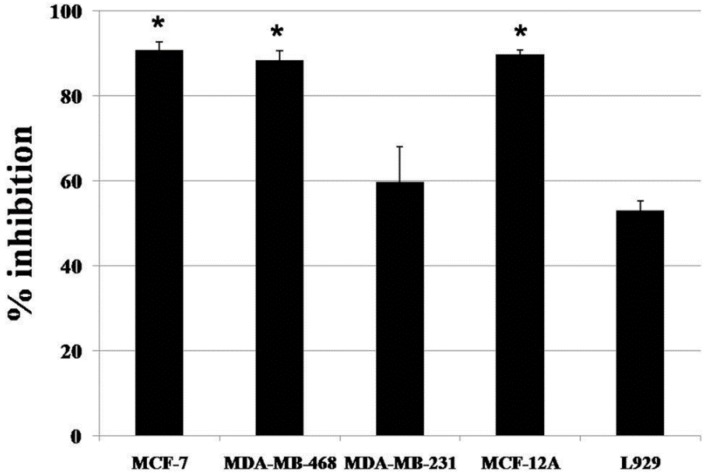
The effect of 5 μg/mL Fraction CE on MCF-7, MDA-MB-468, MDA-MB-231, MCF-12A and L929 cells after an exposure time of 72 h. The values are expressed as the means ± S.D. (*n* = 3). * *p* < 0.05 *versus* normal L929 cells.

The methanolic crude extract was fractionated using column chromatography on silica gel. Six fractions (A to F) were obtained and tested for cytotoxic activity on MDA-MB-468 cell lines. Then, five fractions (B to F), which had demonstrated a cytotoxic effect, were selected to test for cytotoxic activity on breast cell lines. Fraction C showed the most cytotoxic effect on the three breast cancer cell lines, MCF-7, MDA-MB-468 and MDA-MB-231, with IC_50_ values of 2.72 ± 0.03 μg/mL, 3.77 ± 0.43 μg/mL and 4.03 ± 0.88 μg/mL, respectively. The IC_50_ values of the five fractions are shown in Table 2. Then, Fraction C was tested for cytotoxic activity on normal cell lines (MCF-12A) and showed an IC_50_ value of 13.69 ± 2.36 μg/mL. Next, Fraction C was separated into seven fractions (CA to CG) and the cytotoxicity tests repeated with these lines. Fraction CE exhibited a strong effect on MCF-7, MDA-MB-468 and MCF-12A with IC_50_ values of 2.69 ± 0.09 μg/mL, 2.97 ± 0.15 μg/mL and 2.91 ± 0.12 μg/mL, respectively. Surprisingly, Fraction CE demonstrated less toxicity to normal L929 cells with an IC_50_ value of 4.17 ± 0.77 μg/mL (Table 3). As shown in Figure 2, treatment with 5 μg/mL of Fraction CE for 72 h resulted in significant increase in inhibition of cell proliferation on MCF-7, MDA-MB-468 and MCF-12A cells, as compared with L929 cells (*p* ˂ 0.05).

**Table 2:** The IC_50_ values of six fractions isolated from the methanolic crude extract.

Compound	IC_50_ Value ± SD (μg/mL)			
	MCF-7	MDA-MB-468	MDA-MB-231	MCF-12A
Fraction B	10.46 ± 1.28	12.90 ± 1.64	17.54 ± 1.72	ND
Fraction C	2.72 ± 0.28	3.77 ± 0.43	4.03 ± 0.88	13.69 ± 2.36
Fraction D	7.09 ± 0.13	10.16 ± 1.00	20.45 ± 0.48	ND
Fraction E	4.37 ± 1.05	7.05 ± 2.76	13.48 ± 1.65	ND
Fraction F	15.53 ± 0.15	26.52 ± 0.61	46.69 ± 6.84	ND

ND = not determined.

**Table 3:** The IC_50_ values of seven fractions isolated from Fraction C.

Compound	IC_50_ Value ± SD (μg/mL)				
	MCF-7	MDA-MB-468	MDA-MB-231	MCF-12A	L929
Fraction CA	>80	47.21 ± 4.51	>80	ND	>80
Fraction CB	61.70 ± 6.61	23.10 ± 1.99	35.97 ± 0.54	ND	>80
Fraction CC	>80	58.56 ± 2.50	>80	ND	>80
Fraction CD	>80	22.04 ± 2.57	45.03 ± 5.27	ND	>80
Fraction CE	2.69 ± 0.09	2.97 ± 0.15	3.98 ± 0.12	2.91 ± 0.15	4.17 ± 0.77
Fraction CF	25.95 ± 3.24	26.62 ± 4.03	32.68 ± 2.09	ND	55.49 ± 0.91
Fraction CG	>80	>80	>80	ND	>80

ND = not determined.

### 3.4. The Fractions CE Induced DNA Fragmentation on Breast Cancer Cell Lines

**Figure 3:**
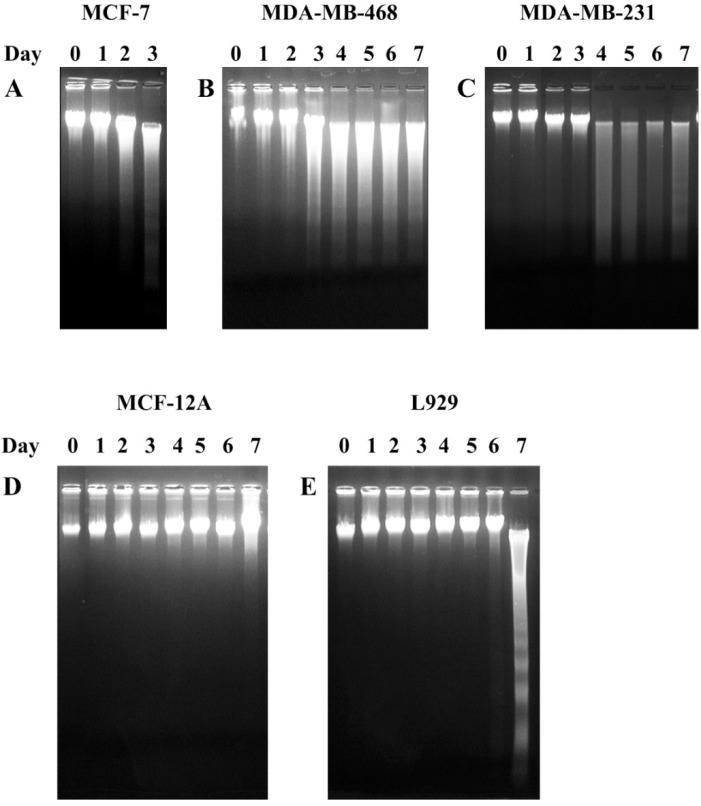
Analysis of DNA fragmentation induced by Fraction CE of *P. cubeba* in five cell lines. Cells were treated for seven days with Fraction CE, and DNA fragmentation was assessed by 1.5% agarose gel electrophoresis and ethidium bromide staining. Fraction CE was used to treat (**A**) MCF-7; (**B**) MDA-MB-468 (**C**) MDA-MB-231; (**D**) MCF-12A and (**E**) L929 with 2.69 μg/mL, 2.97 μg/mL, 3.98 μg/mL, 2.91 μg/mL and 4.17 μg/mL, respectively. The data are representative of three independent experiments carried out under the same conditions.

A DNA fragmentation assay was used to determine whether the action of Fraction CE was associated with apoptosis or not. As shown in Table 3 and Figure 2, Fraction CE was strongly effective on three cell types. In this experiment, all tested cell lines were incubated with Fraction CE at the IC_50_ concentration. DNA fragmentation was found on MCF-7, MDA-MB-468, MDA-MB-231 and L929 cells at 2, 3, 4 and 7 days after exposure (Figure 3). In the MCF-7 cells, the ladder pattern was observed for a maximum of 3 days because all cells died. However, the formation of a DNA ladder was not observed in the MCF12A cells. Therefore, the cytotoxic effect of Fraction CE was selective for breast cancer cell lines and mediated through the induction of apoptosis.

## 4. Discussion

In our study, we used methanol and dichloromethane to extract plant compounds because these two solvents have different polarities. Methanol has a high polarity, while dichloromethane has a low polarity. Therefore, extraction with solvents of different polarities should give different substances. In the extraction procedure, methanolic and dichloromethane extracts were dried on a rotary evaporator until the extracts become solid, then the extracts were dissolved in DMSO. DMSO was used as the control group, and the IC_50_ value from this group was set as the background. Stock solutions of the extracts were prepared in DMSO at high concentrations (approximately 100 mg/mL) to make sure that the final concentrations of DMSO were not more than 0.2% and not toxic to the cells. Therefore, we can conclude that any cytotoxic effects were due to the compound and not the solvent. Moreover, our results demonstrated that the methanol extract was more effective than the dichloromethane extract, in terms of cytotoxic properties. However, Nahak and Sahu reported that the ethanol extract of *P. cubeba* had a higher antioxidant activity than both methanol and aqueous extracts and was also higher than an ethanol extract of *P. nigrum* [13]. Phenolic compounds, such as phenolic acids, flavonoids, quinones, coumarins, lignans, stilbenes and tannins, are rich in antioxidant properties [20,21]. These antioxidant compounds possess anti-inflammatory, anti-atherosclerotic, anti-tumor, anti-mutagenic, anti-carcinogenic, anti-bacterial and anti-viral activities [22,23,24]. In addition, Cai and co-worker reported that total phenolic content of Chinese medicinal plants showed a positive significant linear relationship with antioxidant activity [25].

Methanolic crude extract was separated by column chromatography and gave six fractions (A to F). Fraction C exhibited the most cytotoxic effect on breast cancer cell lines. Therefore, Fraction C was separated into seven fractions (CA to CG). Finally, Fraction CE demonstrated the strongest cytotoxic effect on three breast cancer cell lines. ^1^H-NMR profiles of these most effective extracts of the methanolic crude extracts, Fractions C and CE, showed that these were long chain hydrocarbons (Figure 1). In a previous study, the percentage of essential oil of some *Piper* species has been reported, for example 1.6% of *P. attenuatum* berries, 0.75% of *P. attenuatum* leaf, 1.0% of *P. nigrum* leaf and 14.5% of *P. cubeba* berries. *P. cubeba* berry oil contains 32% monoterpenes, 68% sesquiterpenes and high polar compounds. The major sesquiterpene hydrocarbons are β-elemene (7%) and β-cubebene (6%). The major monoterpene hydrocarbon is β-pinene (18%) [26]. However, we cannot conclude that our long chain hydrocarbons were an essential oil, because these were an unpurified fraction and need to be further purified to obtain the pure compound that has the anti-cancer activity. Essential oils from *Piper demeraranum* and *Piper duckei* inhibit the growth of the promastigote form of *Leishmania amazonensis* and *Leishmania guyanensis* [27]. Yam and co-worker reported that P9605, an extract of *P. cubeba* seeds, suppressed growth on PC-3 prostate cancer cell lines. They hypothesized that this compound could be cubebin [10]. Moreover, Perazzo and colleagues also reported an anti-inflammation activity from the crude aqueous-alcoholic extract of *P. cubeba*, which again they thought was probably due to cubebin [7]. Cubebin is a dibenzylbutirolactone lignan that shows many biological activities [28], including anti-proliferation [29] and anti-inflammation [15].

Here, we used one normal fibroblast, one normal breast and three breast cancer cell lines that had difference characteristics. MCF-12A is the normal breast cell line. This cell is very difficult to culture. Therefore, we designed our study to test only extracts that are known to show high toxicity with breast cancer cell lines and used L929 as a representative of a normal cell. Mouse fibroblast L929 is a normal cell line, which is recommended by international standards for the testing of medical devices [30] and responds more sensitively than primary cells [31]. A plant extract that will act successfully as an anti-cancer drug should kill cancer cells without causing excessive damage to normal cells, such as L929. L929, MCF-7 and MCF-12A cells are p53 wild-types, while MDA-MB-468 and MDA-MB-231 cells are p53 mutations [32]. Furthermore, MCF-7 cells are estrogen-receptor (ER) positive and classified as low-grade and luminal type. MDA-MB-468 and MDA-MB-231 cells are ER negative and classified as high-grade and basal type [33]. Our results showed that Fraction CE had more cytotoxic effects on MCF-7, MCF-12A and MDA-MB-468 than MDA-MB-231, a high-grade cancer. Surprisingly, Fractions CA to CG appeared less toxic in normal fibroblast cells than breast cell lines (Table 3). These results suggest that all fractions were more selective for breast cells than normal cells.

One of the biochemical hallmarks of apoptosis is degradation of DNA by endogenous DNAses, which cut the internucleosomal regions into DNA fragments of 180–200 base pairs. The DNA fragmentation forms a ladder pattern that can be used to distinguish between apoptosis and necrosis [34,35]. This phenomenon can be generally detected by agarose gel electrophoresis, as shown in Figure 3. Here, our results showed that the DNA ladders of MCF-7, MDA-MB-468, MDA-MB-231 and L929 cells treated with Fraction CE were observed within seven days. In contrast, fragmented DNA was not observed in MCF-12A cells. Therefore, DNA ladder formation indicated that the cytotoxic effect of Fraction CE caused inhibition in the growth of breast cancer and normal fibroblast cells through apoptosis. Fraction CE also inhibited growth in normal breast cells, but not to the point of death through apoptosis. In the L929 cells, Fraction CE inhibited cell growth within six days and induced cell death on Day 7 after incubation. Although treated L929 cells showed DNA ladders at Day 7, the incubation time was longer and the dose was higher than in the breast cancer cell lines. Thus, our Fraction CE seemed to be safe for normal cells. However, further experiments are needed to evaluate the specific molecules in the apoptotic pathway. Our previous study showed that *P. nigrum* extract exhibited a cytotoxic effect and induced DNA fragmentation on a breast cancer cell line [19]. In addition, other studies have found that an ethanolic extract of *Mimosa caesalpiniifolia* leaves and an aqueous extract of *Plinia edulis* leaves caused cytotoxicity and induced cell death and DNA fragmentation in MCF-7 cells, via a mechanism that seemed to use the apoptosis pathway [36,37].

## 5. Conclusions

In conclusion, Fraction CE from *P. cubeba* fruit exhibited cytotoxic activity against breast cancer cells and normal breast cells and lower toxicity against normal fibroblast cells. The cytotoxic effect of this fraction inhibited cell growth and appears to have induced apoptosis in MCF-7, MDA-MB-468, MDA-MB-231 and L929 cells. Further purification to obtain a potentially active and pure compound will be undertaken in the future.
